# Knowledge and perception of cardiovascular disease risk among patients with rheumatoid arthritis

**DOI:** 10.1371/journal.pone.0176291

**Published:** 2017-04-24

**Authors:** Sunjoo Boo, Hyunjin Oh, Erika S. Froelicher, Chang-Hee Suh

**Affiliations:** 1 Institute of Nursing Science∙College of Nursing, Ajou University, Suwon, Gyeonggi-do, Korea; 2 College of Nursing, Gachon University, Incheon, Korea; 3 School of Nursing and Medicine, University of California San Francisco, San Francisco, California, United States of America; 4 Department of Rheumatology, Ajou University School of Medicine, Suwon, Gyeonggi-do, Korea; Nagoya University, JAPAN

## Abstract

Patients with rheumatoid arthritis are at increased risk for cardiovascular disease. The prerequisites for reducing the risk of cardiovascular disease are adequate levels of knowledge and being aware of the risk. In this study, the levels of knowledge about cardiovascular disease among patients with rheumatoid arthritis and the perception were evaluated in relation to their actual 10-year risk of cardiovascular disease. This cross-sectional study of 200 patients with rheumatoid arthritis was conducted in a university-affiliated hospital in South Korea. The patients’ actual risk of cardiovascular disease was estimated using the Framingham Risk Score. The most common risk factor was physical inactivity, with 77% of the patients not engaging in regular exercise. The patients lacked knowledge about the effects of physical inactivity and anti-inflammatory medication on the development of cardiovascular disease. Misperceptions about the risk of cardiovascular disease were common, i.e., 19.5% of the patients underestimated their risk and 41% overestimated. Hypertension, diabetes, obesity, and smoking were the most prevalent among the patients who underestimated their risk, and these same patients had the lowest level of knowledge about cardiovascular disease. This study demonstrated the rheumatoid arthritis patients’ lack of knowledge about the effects of physical inactivity and anti-inflammatory medications on the development of cardiovascular disease, and their misperception of cardiovascular risk was common. As a preventive measure, educational programs about cardiovascular disease should be tailored specifically for patients with rheumatoid arthritis, and behavioral interventions, including routine exercise, should be made available at the time of diagnosis.

## Introduction

Rheumatoid arthritis (RA) is a chronic, progressive, and disabling autoimmune disease that affects about 2% of the Korean population [1]. Patients with RA have significantly increased risk of cardiovascular disease (CVD) than general population [2]. They have significantly higher risk of myocardial infarction and sudden cardiac death than those without RA of the same gender and age [3]. The exact causes of this disparity are unclear, but the systemic inflammation associated with RA [4, 5] and medications used to treat RA [6, 7] are likely contributors. Early detection of CVD risk and efforts to prevent the disease are of great importance in this population. The recently updated version of clinical guidelines for preventing CVD specifies RA as a major CVD risk factor and recommends screening patients with RA for CVD risk [8, 9]. Such screening has been shown to benefit patients and reduces costs [10].

Evidence-based clinical guidelines are available to screen patients for CVD risk and classify asymptomatic individuals into low-, moderate-, and high-risk categories, with specific empirically validated risk reduction plans for each subgroup [9, 11, 12]. Risk reduction through improved control of traditional CVD risk factors and therapeutic lifestyle changes is a central strategy for preventing CVD. Studies confirming reductions in morbidity and mortality by controlling CVD risk factors are limited, but recent studies have shown that regular exercise improves fitness and reduces the risk of CVD [13, 14]. Regular physical activity has been shown to substantially reduce the symptoms of RA [15]. Therefore, patients with RA should be counseled about their risk for CVD and encouraged to change their lifestyles to reduce the risk.

Motivating asymptomatic individuals to change their lifestyles to reduce future risk may be challenging. Therefore, it is important to determine what motivates patients with RA to develop healthier lifestyles to reduce risk for CVD. Decisions must be made concerning which people should be the focus of direct risk reduction efforts. Evidence shows that adequate knowledge of CVD risk factors is an essential prerequisite to behavioral changes, but knowledge alone often is insufficient for eliciting preventive actions [16–18]. The likelihood of adopting healthy behaviors may be greater if people perceive themselves at increased risk for CVD [19]. For instance, individuals who perceive themselves to be at risk are more likely to visit healthcare providers and seek screening tests [20, 21].

However, evidence suggests that mismatches are common between actual and perceived risk [22, 23]. Misperceptions of their own CVD risk, whether underestimated or overestimated, might hinder prevention, early detection, and management of CVD. Evaluating patients’ risk perception and making them aware of the actual risk may be prerequisites for effective CVD prevention. However, little is known about the perception of CVD risks among patients with RA in South Korea. Thus, the main objective of this cross-sectional study was to evaluate the levels of knowledge of CVD among patients with RA and to compare their perceived and actual CVD risks. In addition, we valuated underestimation, overestimation, and concordance between perceived and actual risk for CVD, and we described the characteristics of people in each of the three groupings.

## Materials and methods

### Study design, sample, and procedure

A cross-sectional study was conducted in patients with RA in an outpatient clinic of an academic medical center in South Korea. The hospital is an academic medical center with more than 1,100 patients’ beds, and there are approximately 1,000 outpatients with RA. The IRB of Ajou University Hospital approved this study before any patients were recruited (AJIRB-SBR-SUR-15-288). Subjects were eligible to participate in the study if they had been diagnosed and treated for any clinical status of RA and were free of CVD. CVD in this study was defined as a self-reported history of heart attack, angina pectoris, or stroke. Potential subjects were approached while they were waiting for their routine follow-up appointments with their physicians. They were informed about the purpose of the research and the voluntary and anonymous nature of the study. After providing written informed consent, they were asked to fill out the questionnaires. Subjects were encouraged to ask for assistance, if needed. A small gift with a value of $10 was given to the respondents. Of 202 patients who completed the survey, two had missing data on several study variables, and therefore those two were excluded, yielding a final sample of 200 patients with RA in the study. Data were collected in December 2015.

### Measurements

#### Demographics

Demographics, such as gender, age, and educational level were self-reported.

#### CVD knowledge

The subject’s knowledge about CVD was measured with the Heart Disease Fact Questionnaire-Rheumatoid Arthritis (HDFQ-RA) [24]. It is a 13-item questionnaire with two domains, i.e., eight questions about traditional CV risk factors and five questions concerning CVD aspects specific to RA. Subjects were asked to answer ‘yes’ or ‘no’ to each question. One point was awarded for each correct answer, so the scores could range from 0 to 13. The English questionnaires were translated into Korean by two translators and differences were reconciled. The reconciled version was translated back into English by an independent, bilingual translator. The new English version was compared with the original English version to check for any loss of meaning. Content validity was confirmed by two rheumatologists and two registered nurses with Ph.D. degrees who specialized in caring for patients with RA.

#### Cardiovascular risk factors and actual CVD risk

The CVD risk factors identified in this study were hypertension, dyslipidemia, diabetes, parental history of CVD, obesity, cigarette smoking, and physical inactivity. Subjects with a systolic/diastolic blood pressure (BP) of 140/90 mm Hg or more or who were taking anti-hypertensive medications were categorized as hypertensive. Diabetes was defined as a self-report of diagnosed diabetes, the use of glucose-lowering medications, or a fasting blood glucose level of 126 mg/dL or more. Levels of glucose and blood lipids were abstracted from the medical charts. Parental histories of CVD were reported by subjects. Body mass index (BMI, kg/m^2^) was calculated with self-reported height and weight. Asians, compared to Caucasians of the same sex, age, and BMI, have a higher percentage of body fat and more centralized fat distribution; Thus the Asian-Pacific region of World Health Organization (WHO) proposed revised BMI cut-points for Asians: overweight as BMI ≥23 kg/m^2^ and obesity as BMI ≥ 25 kg/m^2^ for both men and women [25]. Although the BMI of 23–<25 kg/m^2^ is considered to be normal in Caucasians, an epidemiological study with 133,740 Korean men and women showed that Koreans with BMIs of 23–<24 kg/m^2^ and 24–<25 kg/m^2^ have respectively a 1.97 and 2.01 fold significantly increased risk for having CVD compared to those with a BMI of 18–<19 kg/m^2^ [26]. In this study, those with BMIs of 23 kg/m^2^ or greater were categorized as overweight or obese based on the obesity criteria for Asians [25]. Histories of renal diseases and smoking habits were self-reported. Subjects were asked to recall the frequency and duration of moderate-intensity regular physical activity engaged in for at least 10 minutes in the past 7 days. Those engaging in at least three times per week of physical activity were considered to be physically active in this study.

Based on guidelines provided by the American Heart Association, actual CVD risk was estimated based on the number of CVD risk factors and the Framingham Risk Score (FRS) [11]. The risk factors were parental history of CVD and the following four CVD risk factors included in the FRS, i.e., 55 or older, BP ≥ 140/90 mmHg or taking anti-hypertensive medication, low levels of HDL-C (<40 mg/dL for men; < 50 mg/dL for women), and current smoking. For those with at least one risk factor, the 10-year CVD risk was estimated using the FRS. The risk estimate was multiplied by 1.5 when a subject met at least two of the following criteria, i.e., more than a 10-year duration of disease, rheumatoid factor (RF) or anti-cyclic citrullinated peptide (anti-CCP) positivity, and presence of extra-articular disease [12]. Diabetes is considered to be equivalent to CVD for risk classification purposes, so they were classified into the high risk group. These steps allowed us to classify the subjects into low (no CVD risk factors), moderate (at least one CVD risk factor and an FRS < 10%), and high risk for developing CVD (at least one CVD factor and an FRS ≥ 10% or those with diagnosed diabetes).

### Perceived CVD risk

One question concerning the likelihood of having CVD in the next 10 years was used to measure the participants’ perceived risk for CVD. Possible response categories were very low, low, moderate, high, or very high; ‘very low’ and ‘low’ responses were combined as ‘low,’ whereas ‘very high’ and ‘high’ responses were combined as ‘high’ so they could be compared with the actual CVD risk in this study.

#### Disease features

Disease features were measured with the Multidimensional Health Assessment Questionnaire (MDHAQ) [27], which includes assessments of physical function, psychological distress, perceived general health, and severity of symptoms of RA, such as pain and fatigue. Higher scores indicate worse levels of physical function, psychological distress, and general health status and more severe levels of pain and fatigue. The MDHAQ has been validated for Korean Patients with RA [28]. RA-related laboratory test results, i.e., C-reactive protein (CRP), RF, and anti-CCP antibody, were obtained from the medical charts.

### Statistical analyses

Statistical analysis was done using SPSS 22 (SPSS statistics IBM Corp. Armonk, NY, USA). Characteristics of the subjects and their CVD risk factors were presented with descriptive statistics. The level of overall CVD knowledge was summarized with mean and median values. The rates at which the subjects answered each knowledge question correctly were presented as percentages. Perceived and actual CVD risks were compared with cross-tabulations and the level of agreement between them was presented with a kappa statistic. Based on the cross-tabulation, subjects were placed into one of three categories, i.e., correspondence (perceived CVD risk agrees with the actual CVD risk), underestimation (perceived themselves to have lower CVD risk than the actual risk), or overestimation (perceived themselves to have higher CVD risk than the actual risk). A series of one way ANOVA and χ^2^-tests (or Fisher’s exact test, when appropriate) were performed to determine any differences in the characteristics of the subjects in the three groups and their CVD risk factors on the agreement between perceived and actual CVD risk. The level of significance was set at 0.05.

## Results

The characteristics of the subjects are presented in Table 1. Most were women (90.5%), and the mean age was 52.6 (± 7.9). The average age at diagnosis of RA in these subjects was 46.8 (± 8.9), and the average duration of the disease was 6.8 (± 5.3) years. The physical functional score was 0.95 out of 10 on average, meaning that the subjects were able to perform most of their daily physical tasks without difficulty. The mean scores for the pain and fatigue related to RA were 3.62 (±2.62) and 4.79 (±2.71) out of 10, respectively.

**Table 1:** Characteristics of participants (*n* = 200).

Characteristics	Number (%) or Mean ± SD	
Gender (female)	181	(90.5)
Age (years)	52.61	± 7.97
Education (college or above)	59	(29.5)
Disease duration (years)	6.81	± 5.32
Age at diagnosis of RA	46.80	± 8.94
Positive RF	97	(48.5)
Positive anti-CCP antibody	64	(32.0)
Extra-articular disease (yes)	50	(25.0)
Morning stiffness (yes)	143	(71.5)
Physical function (0~10)	0.95	± 1.30
Psychological distress (0~10)	2.76	± 2.56
Pain (0~10)	3.62	± 2.62
Fatigue (0~10)	4.79	± 2.71
Perceived general health (0~10)	4.86	± 2.34
Hypertension	78	(39.0)
Taking antihypertensive medication	51	(25.5)
LDL-C ≥ 130 mg/dL	32	(46.0)
Low HDL-C	43	(21.5)
TC/HDL-C	3.12	± 0.86
Taking lipid lowering medication	33	(16.5)
Diabetes	14	(7.0)
Taking insulin or antidiabetic drug	10	(5.0)
BMI ≥ 23 kg/m^2^	86	(43.0)
Current smoker	10	(5.0)
Physical inactivity	154	(77.0)
Parental history of CVD	36	(18.0)
Past history of renal disease	10	(5.0)

SD: standard deviation; RA: rheumatoid arthritis; RF: rheumatoid factor; anti-CCP antibody: anti-cyclic citrullinated peptide antibody; Hypertension refers to BP ≥ 140/90 mm Hg or on antihypertensive medication; TC: total cholesterol; LDL-C: low density lipoprotein cholesterol; HDL-C: high density lipoprotein cholesterol; Low HDL-C refers to HDL-C < 40 mg/dL for men and HDL-C < 45 mg/dL for women; TC: total cholesterol; Diabetes refers to fasting blood glucose ≥ 126 mg/dl or taking glucose lowering medications; BMI: body mass index; Physical inactivity refers to physical activity less than 3 times/week; CVD: cardiovascular disease; CVD refers to self-reported diagnosed heart attack, angina pectoris or stroke.

The most common CVD risk factor found in this sample was physical inactivity, with 77% not engaging in moderate physical activity three or more times per week. Forty-three percent of subjects were categorized as being overweight or obese. Among the subjects, 39% had hypertension, and about 25% were taking anti-hypertensive medications. The ratio of TC/HDL-C was 3.12 on average, and 16.5% were receiving lipid-lowering therapy. Diabetes was present in 7%.

The level of CVD knowledge is summarized in Table 2. The average overall knowledge score was 9.93 (±1.77) out of 13. Most were unaware of the effects of exercise (question #6) and anti-inflammatory medications (question #12) on the development of CVD; however, the relationships between blood pressure, lipids, and CVD were well known. The subjects in this study were less aware of the correct answers to questions 6, 9, and 10 than the subjects in John et al.’s study [24].

**Table 2:** Knowledge of CVD in patients with RA free of CVD (*n* = 200).

Knowledge of CVD	Mean (SD)	Median
Overall knowledge Score (0~13)	9.93 (1.77)	10.00
Questions	% getting it correct	John et al. (24)
1. A person always knows when they have heart disease.	81.0	73.8
2. A person who smokes is more likely to develop heart disease	90.5	86.9
3. Keeping blood pressure under control will reduce a person’s chance of developing heart disease.	91.5	89.2
4. A person with high cholesterol is more likely to develop heart disease.	91.5	90.8
5. If your good cholesterol (HDL) is high you are more likely to develop heart disease.	73.0	33.1
6. Only exercising in gym or in an exercise class will lower a person’s chance of developing heart disease.	35.5	80.8
7. Eating fatty foods does not affect blood cholesterol levels.	86.5	82.3
8. A person with diabetes is more likely to develop heart disease.	83.5	45.4
9. A person with rheumatoid arthritis can reduce their chance of heart disease by keeping their weight under control.	72.0	89.2
10. A person with rheumatoid arthritis can reduce their chance of heart disease by stopping smoking.	79.5	90.8
11. People with rheumatoid arthritis should not exercise because it can damage their joints.	87.5	84.6
12. Anti-inflammatory medications, such as diclofenac or ibuprofen, taken by patients with rheumatoid arthritis may increase the chance of heart disease.	49.5	12.3
13. Having lots of inflammation (‘flares’) of rheumatoid arthritis adds to the increased chance of heart disease.	72.0	23.1

RA: rheumatoid arthritis; CVD: cardiovascular disease; SD: standard deviation.

Cross-tabulation of actual CVD risk and perceived CVD risk is presented in Table 3. Overall, 39.5% of subjects correctly perceived their CVD risk. However, 60.5% misjudged their risk, with 19.5% underestimating their CVD risk and 41% overestimating it. The agreement between perceived and actual risk was low with the kappa statistic of 0.16.

**Table 3:** Perceived risk and actual risk for CVD in patients with RA free of CVD (*n* = 200).

		Actual risk, *n* (%)*				Agreement, *n* (%)			kappa
		Low	Moderate	High	Total	Correspondence	Underestimation	Overestimation	
Perceived risk									
	Low	15 (26.8)	21 (17.5)	2 (8.3)	38 (19.0)	79 (39.5)	39 (19.5)	82 (41.0)	0.16
	Moderate	32 (57.1)	58 (48.3)	16 (66.7)	106 (53.0)				
	High	9 (16.1)	41 (34.2)	6 (25.0)	56 (28.0)				
	Total	56 (100.0)	120 (100.0)	24 (100.0)	200 (100.0)				

* Actual CVD risk was estimated using the Framingham Risk Score. RA: rheumatoid arthritis; CVD: cardiovascular disease.

Further stratification of the cross-tabulation by subjects’ characteristics is presented in Table 4. Those who underestimated their CVD risk were more likely to be men, older, and diagnosed with RA at a later age. They also lacked knowledge about CVD, and the prevalence of hypertension, diabetes, obesity, and smoking was greater than in the other groups. Those who were experiencing severe symptoms of RA, such as morning stiffness and fatigue, overestimated their risk for developing CVD in the future.

**Table 4:** Characteristics of participants by level of agreement between perceived and actual CVD risk (*n* = 200).

Variables	Correspondence (*n* = 79)		Underestimation (*n* = 39)		Overestimation (*n* = 82)		F or χ^2^	*p*	
	Number (%) or Mean ± SD								
Gender (female)	72	(91.1)	29	(74.4)	90	(97.6)	16.611	.000	
Age (years)	53.14	± 8.32	55.90	± 7.46	50.54	± 7.31	6.622	.002	
Education (≥ College)	22	(27.8)	9	(23.1)	28	(34.1)	1.728	.421	
Disease duration (years)	6.09	± 4.63	7.77	± 6.52	7.05	± 5.29	1.449	.237	
Age at diagnosis of RA	47.94	± 9.01	49.44	± 9.48	44.46	± 8.06	5.355	.005	
Positive RF	42	(53.2)	19	(48.7)	36	(43.9)	1.383	.501	
Positive anti-CCP antibody	38	(55.9)	9	(28.1)	17	(27.0)	13.524	.001	
Extra-articular disease (yes)	19	(24.1)	7	(17.9)	24	(29.3)	1.869	.393	
Morning stiffness (yes)	53	(67.1)	22	(56.4)	68	(82.9)	10.367	.006	
Physical function	0.85	± 1.18	0.92	± 1.17	1.04	± 1.46	0.445	.642	
Psychological distress	2.46	± 2.45	2.91	± 2.69	2.98	± 2.62	0.882	.416	
Pain	3.33	± 2.47	3.72	± 2.75	3.85	± 2.70	0.823	.440	
Fatigue	4.34	± 2.58	4.73	± 3.04	5.25	± 2.64	2.326	.100	
Perceived general health	4.49	± 2.27	5.04	± 2.56	5.14	± 2.26	1.721	.182	
Hypertension	31	(39.2)	23	(59.0)	24	(29.3)	9.807	.007	
TC/HDL-C	3.04	±.78	3.32	± 1.07	3.09	±.80	1.422	.244	
Diabetes	5	(6.3)	9	(23.1)	-	-	21.711	.000	
BMI ≥ 23 kg/m^2^	30	(38.0)	23	(59.0)	33	(40.2)	6.129	.045	
Current smoker	3	(3.8)	5	(12.8)	2	(2.4)	6.394	.046	*
Physical inactivity	60	(75.9)	30	(76.9)	64	(78.0)	0.100	.975	
Parental history of CVD	12	(15.2)	7	(17.9)	17	(20.7)	0.837	.699	
Past history of renal disease	4	(5.1)	1	(2.6)	5	(6.1)	0.561	.913	*
CVD Knowledge	10.00	± 1.63	9.23	± 1.80	10.21	± 1.82	4.240	.016	

*Fisher’s exact test.

CVD: cardiovascular disease; n: total sample; SD: standard deviation; RA: rheumatoid arthritis; RF: rheumatoid factor; anti-CCP antibody: anti-cyclic citrullinated peptide antibody; Hypertension refers to BP ≥ 140/90 mm Hg or on antihypertensive medication; TC: total cholesterol; HDL-C: high density lipoprotein cholesterol; Diabetes refers to fasting blood glucose ≥ 126 mg/dl or taking glucose lowering medications; BMI: body mass index; Physical inactivity refers to less than 3 times/week.

## Discussion

Patients with RA are twice as likely to develop CVD as those without RA [2]. Compared to the general population of Korean women [29], the prevalence of traditional CVD risk factors, such as hypertension and diabetes, was high in this study’s sample, as was the proportion of those at increased risk of CVD. In this study, 12% of the subjects with RA were categorized as having high risk for CVD, and 60% and 28% were categorized as having moderate risk and low risk, respectively. In a study using a representative sample of Korean women nationwide, 7.9% had high risk for CVD, 20.5% had moderate risk, and 71.6% had low risk [29]. Even though the average age of the subjects was about five years older than those in Boo and Froelicher’s study [29] and about 10% of the people in the study were men, the excessive risk of CVD that was found in this study may be partially attributable to RA. Risk reduction through better control of modifiable CVD risk factors and therapeutic changes in lifestyle are critically important for preventing CVD in this group. Levels of CVD knowledge and an accurate risk perception affect people’s willingness to change to healthy lifestyles. In this study, we examined the knowledge and risk perception of CVD in patients with RA in Korea to get some insight in designing evidence based interventions to reduce their increased risk.

In this study, subjects were generally well aware of traditional CVD risk factors. Majority were identified hypertension (91.5%), dyslipidemia (91.5%), smoking (90.5%) and diabetes (83.5%) as CVD risk factors. Even though the differences in measures make it hard to simply make comparisons of levels of CVD knowledge to those of general Koreans, it seems like that the subjects of this study are more knowledgeable about traditional CVD risk factors than previously reported. In a study of random sample of 1,304 Korean women (mean age of 50.24), 77.1% and 38.1% identified hypertension and diabetes as risk factors for CVD, respectively [30]. A study of Koreans aged 60 years or older reported that 78.4% and 47.1% were aware the effects of hypertension and diabetes on the development of CVD, respectively [31].

The overall average score of CVD knowledge in our subjects was 9.93 (± 1.76) out of 13. Among 13 items, Korean patients with RA got higher rates of correct answers in 10 items than U.S. patients [24]. Although it is difficult to compare scores of patients across different studies, the knowledge scores show that the current participants had a higher level of knowledge about CVD than those in John et al.’s study [24]. This may be related to the differences in their demographic characteristics. Our subjects were about 12 years younger and had higher education levels than those in John et al.’s study [24]. The three items that had lower correct rates than the subjects in John et al.’s study [24] were items 6, 9, and 10, i.e., physical activity, weight loss, and smoking cessation. Especially for item 6, the percentage of subjects who answered correctly was the lowest, with about 65% of the subjects indicating that only working out in the gym or in an exercise class would lower their risk for CVD. This is a significant concern, because most of the subjects in this study were physically inactive despite no difficulties in carrying out daily physical tasks. However, regular, even low intensity exercise is an integral part of long-term CVD risk reduction as well as better control of RA symptoms.

Although only 5% of subjects were current smokers, they were less aware that quitting smoking reduces their chance of getting heart disease. As shown in Table 2, subjects were well aware that smoking is a risk factor for CVD (question 2, 90.9%), but they were less aware of the beneficial effects of quitting smoking (question 10, 72.1%). Among smokers, only 40% were correctly answered for the question 10 (data not shown). Educational efforts should focus on the factors that affect the development of CVD and on ways to reduce the increased CVD risk, with the benefits of risk reduction that can be obtained by behavioral changes. In addition, the level of knowledge about RA-specific CVD risk generally was lower than that of generic CVD knowledge. An earlier study focused on the unmet educational needs concerning the CVD aspects of RA [32]. Lack of awareness and non-adherence to CVD prevention guidelines by healthcare providers could be a possible reason for RA patients’ lack of knowledge about RA-specific CVD. Future research is warranted to evaluate healthcare providers’ awareness of and adherence to CVD prevention guidelines. Tailored education programs regarding CVD risk specific to RA should be developed and delivered to patients with RA at the time of diagnosis so that appropriate risk reduction efforts can be implemented.

A mismatch between perceived and actual CVD risk seemed to be common in patients with RA (Table 3), and this was consistent with previous reports of other populations [23, 33]. Approximately 20% of patients with RA underestimated their CVD risk, and 41% overestimated it. Since actual CVD risk does not necessarily match with an individual’s risk perception, it is worth considering the specific characteristics of patients with RA who misperceive their CVD risk. Regrettably, hypertension, diabetes, obesity, and smoking were the most prevalent among the subjects who underestimated their CVD risk, and these same subjects had the lowest level of CVD knowledge (Table 4). Patients who were men, older, and had been diagnosed RA at an older age underestimated their CVD risk compared to their counterparts. This is consistent with previous studies in which women were reported to have a higher perceived CVD risk than men [33, 34], and Korean men tend to have a more optimistic perception of their health than Korean women [34, 35]. Additional research should be conducted to determine whether such gender-associated differences in risk perception among patients with RA affect health behaviors or CVD health outcomes. For the prevention of CVD, patients with RA need to be aware of their CVD risks and follow risk reduction strategies. An individual’s CVD risk perception is related to various activities and affects personal decisions and healthy behaviors [36]. When patients with a high risk of CVD have optimistic perceptions of their CVD risk, they might overlook the importance of engaging in preventive activities. That is why we need to more focus on patients’ underestimation of their CVD risk.

Patients with morning stiffness might have a poor perception of their health and overestimate their CVD risk. Patients with RA experience various general and muscular symptoms, such as functional limitations, pain, and fatigue. Although there is no statistical difference, they may perceive their general health to be too poor to correct, and their perception of their CVD risk might be overestimated. In the literature, it has been shown that an individual’s perception of her or his general health [35] and level of knowledge [33] are related to the willingness to engage in healthy behaviors. According to the Health Belief Model, knowledge and perception of increased risk are necessary conditions for behavioral changes [37]. Generally, it is accepted that people who overestimate their actual CVD risk are more likely to engage in beneficial behaviors, such as smoking cessation, weight loss, physical activity, and medication adherence [18, 38]. The patients with RA in this study who overestimated their CVD risk were non-smokers and had optimal weights. However, there was no statistical difference for physical inactivity.

CVD knowledge was a factor that influenced the underestimation of CVD risk in patients with RA. Adequate knowledge about CVD risk is an important prerequisite for making appropriate decisions concerning the prevention of CVD [33]. As stated earlier, to encourage behavioral changes, patients must be educated about CVD risk factors and the vulnerability of patients with RA to CVD. In this context, communication about the perception of CVD risk and actual risk factors between patients and healthcare providers is very important, and it is required to improve the accuracy of people’s risk perception and health behaviors.

There are several limitations that should be acknowledged in order to appropriately interpret the results of this study. First, we used the FRS to estimate actual 10-year CVD risk because no culture specific tool was available. Notably, there is evidence that shows that the FRS underestimates the risk of CVD in women [39, 40] and in patients with RA [41]. However, some studies have indicated that the FRS overestimates CVD risk in Asians [42, 43]. When data are available, recalibration of the FRS should be considered to improve its accuracy in this population. Second, self-reported data for extra-articular diseases may affect the study’s findings. The bias would be in the direction of underestimating the true risk. Third, most of the subjects in this study were women. This was, in part, because the incidence of RA is much higher in women. In previous multi-center studies of patients with RA in Korea, about 85–95% of the patients were women [44, 45]. However, given that the incidence of CVD is higher in men than in pre-menopausal women, further studies should oversample men to have parity to enable gender-specific analyses. Nevertheless, this study identified specific CVD risk factors lacked knowledge and showed that there was a mismatch between perceived and actual CVD risk among patients with RA in Korea. By further clarifying the misperception into underestimation and overestimation and examining differences in characteristics of patients and their CVD knowledge according to risk perception, the findings of this study can be helpful in designing more appropriate educational materials for patients with RA and in directing cognitive behavioral interventions to reduce CVD risks.

## Conclusions

This study demonstrated the subjects’ lack of knowledge about the effects of physical inactivity and anti-inflammatory medications on the development of CVD. Patients with RA should know RA-specific risk factors and their increased risk for CVD. Tailored education programs regarding the cardiovascular risk that is specific to patients with RA should be developed and delivered to them at the time of diagnosis.

## Supporting information

S1 File The data file of 200 patients with rheumatoid arthritis. (XLS)
